# Serum, Dietary, and Supplemental Vitamin D Levels and Insulin Resistance in 6294 Randomly Selected, Non-Diabetic U.S. Adults

**DOI:** 10.3390/nu14091844

**Published:** 2022-04-28

**Authors:** Larry A. Tucker

**Affiliations:** College of Life Sciences, Brigham Young University, Provo, UT 84602, USA; tucker@byu.edu; Tel.: +1-801-422-4927

**Keywords:** insulin sensitivity, type 2 diabetes, metabolic, glucose, pancreas, HOMA

## Abstract

The primary aim of this study was to determine the associations between serum, dietary, and supplemental vitamin D levels and insulin resistance in 6294 non-diabetic U.S. adults. A total of 8 years of data from the 2011–2018 National Health and Nutrition Examination Survey (NHANES) and a cross-sectional design were utilized to answer the research questions. Serum vitamin D levels were quantified using high-performance liquid chromatography–tandem mass spectrometry. Dietary and supplemental vitamin D intakes were assessed using the average of two 24 h dietary recalls taken 3–10 days apart. The homeostatic model assessment (HOMA), based on fasting glucose and fasting insulin levels, was employed to index insulin resistance. Demographic covariates were age, sex, race, and year of assessment. Differences in physical activity, body mass index (BMI), cigarette smoking, body weight, season, and energy intake were also controlled statistically. Serum levels of vitamin D differed significantly, and in a dose–response order, across quartiles of HOMA-IR, after adjusting for year, age, sex, and race (F = 30.3, *p* < 0.0001) and with all the covariates controlled (F = 5.4, *p* = 0.0029). Dietary vitamin D levels differed similarly across HOMA-IR quartiles, but to a lesser extent, respectively (F = 8.1, *p =* 0.0001; F = 2.9, *p =* 0.0437). Likewise, supplemental vitamin D levels also differed across the HOMA-IR quartiles, respectively (F = 3.5, *p =* 0.0205; F = 3.3, *p =* 0.0272). With all the covariates controlled, the odds of having insulin resistance were significantly greater for those in the lowest quartile of serum and supplemental vitamin D intake compared to the other quartiles combined. In conclusion, in this nationally representative sample, serum, dietary, and supplemental vitamin D were each predictive of insulin resistance, especially in those with low serum levels and those with no supplemental intake of vitamin D.

## 1. Introduction

The hormone insulin helps to regulate the amount of glucose in the blood. With insulin resistance, cells of the body do not respond normally to insulin. Consequently, glucose is not moved into cells at a healthy rate and blood glucose concentrations increase. As blood glucose levels rise, the body responds by increasing the amount of circulating insulin. Over time, individuals lacking insulin sensitivity develop excess concentrations of insulin in the blood.

Insulin resistance is predictive of a variety of chronic diseases. Atherosclerosis [1] and ischemic heart disease [2] are closely tied to insulin resistance. High blood pressure [3], stroke [4], metabolic syndrome [5], and cardiovascular disease [6] are also related significantly with insulin resistance. Moreover, inadequate insulin sensitivity often leads to type 2 diabetes [7]. 

A number of conditions increase the risk of insulin resistance. Most of the causes are a function of an unhealthy lifestyle. Some of the most common risk factors are physical inactivity [8], obesity [9], smoking [10], and abdominal obesity [11]. Macronutrient intake is also predictive of insulin resistance [12,13]. Although closely linked with insulin resistance, these risk factors tend to be difficult to change, requiring considerable lifestyle adjustments. 

Although macronutrient patterns are predictive of insulin resistance, research also shows that some micronutrients, particularly vitamin D, are related to insulin sensitivity [14,15], as well as type 2 diabetes [16]. Specifically, it appears that adults with low levels of vitamin D are at increased risk of insulin resistance. Given vitamin D levels can be easily influenced and low levels can be remedied with little effort or cost, targeting vitamin D to prevent or reduce insulin resistance is a potentially attractive therapy. 

Over the past few decades, many studies have been conducted to measure the association between vitamin D levels and insulin sensitivity, as shown in the review by Wallace et al. [17]. The literature indicates that mixed results are commonplace [17]. The inconsistent findings could be partly because investigations have differed significantly in their designs, and measurement methods have included a variety of techniques. Additionally, almost all studies in this area have utilized samples of convenience resulting in few generalizable findings. Consequently, the primary objective of this research was to evaluate the connection between serum and dietary levels of vitamin D, including food and supplemental sources, and insulin resistance, in a large sample of randomly selected women and men selected from the U.S. population. Another objective was to measure the impact of 10 covariates, including age, sex, race, year of assessment, BMI, smoking, body weight, physical activity, season, and energy intake, on the association between vitamin D levels and insulin resistance. 

## 2. Materials and Methods

### 2.1. Study Design and Sample

The research questions of the present study were answered using the NHANES database. Comprehensive interviews, blood samples, physical examinations, and questionnaires were employed to collect the NHANES data. Trained technicians performed the various assessments on randomly selected individuals. 

Adults with diabetes or individuals reporting that they took oral medication or insulin to manage their blood glucose levels, or they were determined to have an elevated fasting blood glucose level, were not included in the analyses. Additionally, participants reporting zero energy consumption during either of the dietary assessments were not included in the analyses.

U.S. non-institutionalized civilians were selected randomly using a multi-stage protocol. Adults aged 18–80 years were included in the sample. Census data were used as reference points so that the final results were representative of the U.S. adult population.

The current study utilized NHANES data collected during an 8-year period, 2011–2018. More recent data are not available because the COVID-19 pandemic prevented NHANES from performing the assessments for many months. Each individual who participated in the survey gave written informed consent before any information was collected. The Ethics Review Board for the NCHS authorized the methods and storage of the information [18]. The codes confirming ethical approval for the NHANES data collected from 2011–2018 are: Protocol #2011-17 and Protocol #2018-01.

There were 11,223 individuals in the original NHANES sample, which included participants 12–80 years old. When only subjects 18–80 years of age were included, the sample was 9723. A total of 1769 individuals self-reported having diabetes, taking oral diabetic medication, using insulin, or they were identified as diabetic as a result of the fasting blood glucose test, leaving 7954 participants. The sample was reduced to 7400 adults due to refusal to complete, partial completion, or not consuming any energy (the participant fasted) for the first 24 h dietary recall, or not completing the dietary supplement part of the 24 h recall assessment. The sample was decreased to 6528 for the same issues associated with the second 24 h recall. A total of 178 adults refused to give blood, the phlebotomist could not draw sufficient blood, or some other blood draw problem, resulting in 6350 participants. There were 55 subjects without BMI scores and 1 participant who did not report cigarette use or non-use resulting in a final sample of 6294 adults. 

### 2.2. Instrumentation and Measurement Methods

Serum, dietary, and supplemental levels of vitamin D were the exposure variables for this study. The extent to which they were predictive of insulin resistance was evaluated. Age, sex, race, year of assessment, physical activity, BMI, cigarette smoking, body weight, season, and energy intake, were included as covariates and were controlled statistically. 

#### 2.2.1. Insulin Resistance

HOMA-IR was employed as a marker of insulin resistance. HOMA-IR is the most commonly used variable in the literature to index insulin resistance. Search engines indicate that there are almost 20,000 journal articles including the term “HOMA-IR” or “HOMA.” The HOMA-IR formula is based on fasting concentrations of plasma glucose and insulin levels: insulin (μU/mL) × fasting glucose (mg/dL) ÷ 405. Among the 6294 randomly selected participants, those with HOMA-IR values ≥ the 75th percentile were considered insulin-resistant, consistent with other research [19,20,21]. 

NHANES assigned participants randomly to attend a blood draw session in the morning. They were asked to fast for 9 h. Comprehensive explanations are posted online about the methods employed to gather the fasting glucose and insulin data [22,23,24,25]. 

#### 2.2.2. Serum, Dietary, and Supplemental Vitamin D 

Specific information about specimen collection and processing are explained in the comprehensive NHANES Laboratory Procedures Manual [26]. Detailed quality assurance and quality control information is also discussed in the manual [26]. 

High-performance liquid chromatography–tandem mass spectrometry was utilized for the precise measurement of 25-hydroxyvitamin D3 (25OHD3), 3-epi-25-hydroxyvitamin D3 (epi-25OHD3), and 25-hydroxyvitamin D2 (25OHD2), as described on the NHANES website [27]. For the present investigation, 25OHD2 and 25OHD3 were summed, providing a calculation of total serum vitamin D (nmol/L) [27]. Serum levels of vitamin D were measured from 2011–2016, but not 2017–2018. Consequently, analyses including blood levels of vitamin D were based on 4805 subjects, not 6294. Relationships focused on dietary or supplementary vitamin D and insulin resistance included the sample of 6294 men and women. 

To determine dietary intake of vitamin D, two 24 h dietary recall assessments were performed. The first recall interview occurred in-person. Data for the second interview was collected via telephone 3 to 10 days after the first assessment. The average of the two dietary recalls was used to represent dietary intake of vitamin D in the U.S. 

The dietary evaluations collected comprehensive information about all foods and beverages consumed during the 24 h period prior to the interview (midnight to midnight). Participants who did not consume any calories during either of the dietary assessment periods were not included in the sample. 

A comprehensive 24 h dietary supplement assessment was also administered. Data were collected on all dietary supplements that were ingested from midnight to midnight, including the name and the amount of the supplement consumed.

Two 24 h recall assessments 3 to 10 days apart are sufficient to represent the dietary intake of the population in the United States, given that the sample size of the present study was large. In his book, *Nutritional Epidemiology*, Willett states that one 24 h dietary recall is satisfactory when the sample is of adequate size [28]. He also indicates that “it is statistically most efficient to increase the number of individuals in the sample, rather than to increase the number of days beyond 2 days per individual” (page 55) [28]. Because this investigation included a large sample of randomly selected adults, with each subject completing two 24 h dietary assessments and two dietary supplement assessments, the methods used were suitable to obtain quality estimates of vitamin D intake. 

The professionals who administered the diet recall interviews were carefully trained. Each was a graduate in nutrition or a similar major. Additionally, the individuals conducting the dietary recall interviews were bilingual.

Before collecting data for NHANES, each dietary interviewer participated in an intense one-week training course. Supervised practice interviews were also conducted. Annually, retraining sessions were performed to reinforce correct procedures and techniques. Furthermore, the technicians were evaluated over the data gathering process. 

The dietary recall assessments were computer-based, providing a standard interview protocol, and the interviewers used scripts to guide them. The 24 h recalls followed a multi-pass format, described online [29]. Food probes were used during the dietary interviews that have been employed in previous national studies. 

Multiple real-life objects were utilized to provide participants with representative examples. Specifically, the in-person interviews included different-sized glasses, bowls, dishes, etc. Following the first dietary assessment, participants were given sample dishes and a food model document to take with them to help during the second dietary assessment.

### 2.3. Covariates

A total of 6 classifications were employed by NHANES to distinguish among races: Mexican American, Non-Hispanic (NH) Black, NH White, NH Asian, Other Hispanic, or Other Race/Multi-racial. Race was controlled statistically in this investigation. 

A digital scale was used to measure body weight. During the weighing procedure, subjects wore a standard disposable paper gown, and underwear. Standing height was measured with a stadiometer. The body mass index (BMI) was determined using the weight and height results: weight (kg)/height (m^2^). BMI has been shown to be linearly and consistently associated with insulin resistance [30]. 

Level of physical activity also served as a covariate because of its consistent relationship with insulin resistance [8,31]. An interview was used to gather information about participation in moderate and vigorous physical activities. Total minutes of moderate and total minutes of vigorous activity per week were added together to form the variable, minutes of moderate and vigorous physical activity (MVPA) per week.

Smoking is a good predictor of insulin resistance [10]. Smoking behavior was measured by evaluating the number of cigarettes smoked per day during the past month. Zero cigarettes were recorded for non-smokers and smokers were given values up to a maximum of 95 cigarettes per day. 

Energy intake was measured using the two 24 h dietary recall assessments. Energy intake was recorded in kilocalories (kcal). The average of the two recall records was used to index energy intake. Mean energy intake was used as a covariate. 

Because 8 years of data were used in this study, year of assessment was recorded and used as a covariate. Additionally, because season of the year impacts exposure to sunlight, which influences serum levels of vitamin D, season of assessment was recorded and used as a covariate. Two categories were utilized to define season: warm season (1 May–31 October) and cool season (1 November–30 April). NHANES does not provide more specific season information to minimize the risk of identifying participants based on when data were collected from them. 

### 2.4. Data Analysis

Statistical analyses using NHANES data are unique. Because NHANES employs a 4-stage sampling strategy to select participants, strata, clusters, and individual sample weights must be included in each statistical model so that the findings can be generalized to the U.S. adult population. According to NHANES, the sample weights reflect the unequal probability of selection, nonresponse adjustment, and adjustment to independent population controls [32]. When unequal selection probability is applied, unbiased national estimates result from using the sample weights in statistical analyses [32].

Moreover, although the primary sample size of this study was large (n = 6294), given the multistage sampling scheme used by NHANES, degrees of freedom (df) were based on the number of clusters (121) minus the number of strata (58), resulting in 62 df, instead of 6294 df. Analyses that included serum levels of vitamin D included 47 df, calculated by subtracting 44 strata from 91 clusters. 

HOMA-IR was the outcome variable and there were 3 exposure variables: serum, dietary, and supplemental vitamin D. Four demographic covariates were included: age, sex, race, and year of assessment. Additionally, BMI, smoking, physical activity, body weight, season, and energy intake were included as covariates to decrease their impact on the associations between vitamin D and insulin resistance. To be included in the analyses, participants were required to have complete data. However, serum levels of vitamin D were not available for the 2017–2018 data collection cycle. Consequently, analyses including serum levels of vitamin D were based on 4805 subjects, not 6294.

Two statistical analysis methods were used to assess the connection between the vitamin D exposure variables and HOMA-IR. First, HOMA-IR was divided into quartiles and mean differences in serum, dietary, and supplemental vitamin D were compared across the quartiles using multiple regression and the SAS SurveyReg procedure. Statistical adjustments were made for differences in the covariates utilizing partial correlation. The Least Squares Means (LSMeans) method was utilized to produce adjusted means. Because approximately 73% of the participants did not consume any supplemental vitamin D, the distribution was skewed. Therefore, the cube-root of each value was used. This decreased the SAS skewness index for supplemental vitamin D from 24.5 to 1.9. 

Second, the SAS SurveyLogistic procedure was utilized to calculate odds ratios between the serum and dietary vitamin D variables, each divided into quartiles, and insulin resistance, defined as the highest quartile of HOMA-IR. Odds ratios were adjusted for differences in the covariates. Because roughly 27% of the subjects consumed some supplemental vitamin D, only two supplemental vitamin D categories were used. Specifically, supplement users were compared to adults reporting no supplemental vitamin D intake. To analyze the data, SAS version 9.4 (SAS Institute, Inc., Cary, NC, USA) was used. Alpha was set at <0.05 to define statistical significance, and all the statistical tests were two-sided.

## 3. Results

NHANES employed a multistage random sampling scheme to ensure participants represented the U.S. population. There were 6294 women and men in the investigation. Average age (±SE) of the sample was 45.5 ± 0.4 years. Mean (±SE) HOMA-IR was 2.74 ± 0.05 and mean (±SE) body mass index (BMI) was 28.6 ± 0.15 kg/m^2^. Mean (±SE) serum vitamin D was 70.9 ± 0.98 nmol/L. Average (±SE) body weight was 81.6 ± 0.40 kg and mean (±SE) weekly moderate to vigorous physical activity (MVPA) reported by subjects was 170.8 ± 5.90 min. Mean energy intake was 2109 ± 14 kilocalories (kcal) per day. Lastly, mean dietary vitamin D intake was 4.5 ± 0.08 mcg/day and mean intake of supplemental vitamin D was 12.7 ± 1.18 mcg per day. Table 1 shows the distribution of values across percentiles of the main variables. 

### 3.1. Mean Differences in Vitamin D Levels across Quartiles of HOMA-IR

Table 2 displays the extent to which mean serum concentrations of vitamin D differed across quartiles of HOMA-IR. The relationship was strong and dose-response (F = 30.3, *p <* 0.0001). Specifically, after adjusting for differences in year of assessment, age, sex, and race (model 1), serum levels of vitamin D were lowest in adults who had insulin resistance (HOMA-IR quartile 4) and highest in quartile 1 of HOMA-IR. Controlling for differences in BMI, physical activity, body weight, smoking, season, and energy intake, in addition to the demographic covariates, weakened the association, but it remained significant (F = 5.4, *p =* 0.0029).

Dietary intake levels of vitamin D also differed significantly across the HOMA-IR quartiles. As shown in Table 2, mean concentrations of dietary vitamin D were inversely distributed across the HOMA-IR quartiles, after controlling for the demographic covariates (F = 8.1, *p =* 0.0001) and the other potential confounding variables (F = 2.9, *p =* 0.0437). 

Mean levels of supplemental vitamin D were also inversely distributed across the HOMA-IR quartiles. The lowest supplement levels were found in adults with insulin resistance (HOMA-IR quartile 4) and the highest supplement amounts were reported in adults in quartile 1. Controlling for differences in the covariates had little influence on the mean differences, as shown in Table 2. With only the demographic covariates controlled, differences in supplemental intake were significant (F = 3.5, *p =* 0.0205). After adjusting for all the covariates, the relationship changed little (F = 3.3, *p =* 0.0272). 

The supplement intake distribution was skewed, so the cube-root of each value was used. For serum levels of vitamin D, mean differences between Q2 vs. Q3 (model 1), and Q1 vs. Q3 (model 2), and dietary intake, Q2 vs. Q3 (model 1), and Q1 vs. Q3 (model 2), and supplement intake, Q1 vs. Q2 (models 1 and 2) were borderline significant (*p* > 0.05 and *p <* 0.09). Model 1 included four covariates: year, age, sex, and race. Model 2 included all the covariates: year, age, sex, race, BMI, physical activity, weight, smoking, season, and energy intake. 

### 3.2. Association between Vitamin D and Insulin Resistance Indexed Using Odds Ratios

With both variables conveyed using quartiles, the odds of being insulin-resistant (HOMA-IR ≥ the 75th percentile) based on serum levels of vitamin D are shown in Table 3. Adults in the lowest quartile of serum vitamin D had exactly twice the odds of being insulin-resistant compared to those not in quartile 1. With all the covariates controlled, the odds were lower, but significant. Specifically, those with the lowest serum levels (quartile 1) had 54% greater odds of being insulin-resistant compared to their counterparts (i.e., quartiles 2, 3, or 4). Comparing only the most extreme quartiles of serum vitamin D (Q1 vs. Q4) resulted in 2.81 times higher odds of insulin resistance in those with low serum levels (Q1) versus high levels (Q4), after adjusting for the demographic covariates. With all the covariates controlled (age, sex, race, year, physical activity, BMI, smoking, body weight, season, and energy intake), the odds of having insulin resistance were 67% higher for those with low (Q1) compared to high (Q4) serum vitamin D levels (Table 3). 

The odds of being insulin-resistant (HOMA-IR ≥ the 75th percentile) based on dietary quartiles of vitamin D are shown in Table 4. Comparing adults who reported low intakes of vitamin D (quartile 1) to the other adults (quartiles 2–4) resulted in non-significant odds ratios. However, when those with low intakes (quartile 1) were compared to those with high levels of dietary intake (quartile 4), adults with low intakes had 20% higher odds of being insulin-resistant compared to those in the highest quartile. After adjusting for all the covariates, the relationship was no longer significant (Table 4). 

Approximately 73% of the sample reported no supplement intake of vitamin D. Therefore, participants could not be divided into quartiles or equal-sized categories. Adults reporting no supplement intake were treated as the reference group (ref), and they were compared to adults who reported taking supplemental vitamin D. As shown in Table 5, adults reporting no vitamin D supplement use had 42% greater odds of having insulin resistance compared to their counterparts, with the demographic variables controlled. After adjusting for all the covariates, those with no vitamin D supplement use had 36% greater odds of having insulin resistance compared to those reporting vitamin D supplement intake. 

### 3.3. Relationships among Serum, Dietary, and Supplemental Vitamin D Levels 

The strongest bivariate correlation among the three measures of vitamin D was between supplemental and serum levels of vitamin D. Regression analysis showed that the bivariate correlation between supplemental vitamin D and serum levels was 0.52 (*p <* 0.0001). Between dietary vitamin D and serum levels, the bivariate correlation was 0.08 (*p <* 0.0001), and between supplemental and dietary vitamin D, the correlation was 0.06 (*p =* 0.0009).

## 4. Discussion

The primary goal of this investigation was to determine the associations between serum, dietary, and supplemental vitamin D and insulin resistance, indexed using HOMA-IR, in a large sample of women and men representative of the U.S. population. Age, sex, race, year of assessment, smoking, BMI, physical activity, body weight, season, and energy intake were employed as covariates to help control their influence on the relationships. 

There were five key findings associated with the present investigation: (1) There was a dose–response relationship between serum levels of vitamin D and insulin sensitivity (HOMA-IR). Additionally, individuals in the lowest serum vitamin D quartile had 2.0 times greater odds of being insulin-resistant than adults in quartiles 2, 3, and 4 combined, after adjusting for differences in the demographic variables. (2) There was a dose–response association between dietary intake of vitamin D and HOMA-IR. (3) Individuals displaying the highest insulin sensitivity had the highest supplemental vitamin D intakes, and adults who did not consume any supplemental vitamin D had 36–42% greater odds of being insulin-resistant compared to those who consumed supplemental vitamin D. (4) Serum level of vitamin D was the best predictor of insulin resistance. (5) Supplement intake of vitamin D was a better predictor of serum levels of vitamin D than dietary intake of vitamin D. 

### 4.1. Serum Levels of Vitamin D and Insulin Resistance 

As logic would predict, in the present study, serum levels of vitamin D accounted for differences in HOMA-IR better than dietary or supplemental intakes of vitamin D. Moreover, the relationship between serum levels of vitamin D and insulin sensitivity was dose–response. The more vitamin D detected in the blood, the more insulin-sensitive men and women tended to be. This is because there is less error in the measurement of serum levels of vitamin D than the dietary and supplemental assessments. Additionally, serum levels include the effect of sunlight exposure, a significant contributor to vitamin D levels [33,34].

Multiple correlational studies have detected a significant relationship between serum levels of vitamin D and insulin sensitivity [35,36,37,38,39]. For example, using a sample of 79 individuals with type 2 diabetes and 22 age-matched healthy controls, Jain et al. determined that there was a significant correlation between plasma levels of vitamin D and HOMA-IR (*r* = −0.31, *p =* 0.03) [35]. Additionally, in an investigation by Chen et al., a sample of 1876 adults was studied. Using Spearman correlation, serum levels of vitamin D were inversely correlated with HOMA-IR (*r* = −0.19, *p <* 0.0001) [37]. Similarly, in a sample of 494 working Japanese men, 20–68 years old, HOMA-IR levels were significantly and inversely associated with plasma vitamin D levels divided into quartiles [38]. Finally, in a sub-sample of the Tromso study, 52 subjects with high serum vitamin D were compared to 108 with low serum levels using a hyperglycemic clamp. Those with high serum vitamin D levels had higher insulin sensitivity scores than those with low levels. However, when potential confounders were controlled, the association was nullified [39]. 

Hutchinson et al. studied over 2000 subjects using a cross-sectional design. Participants had varying levels of glucose tolerance impairment. When the sample was divided into quartiles based on serum vitamin D levels, insulin resistance, measured using HOMA-IR, was lower for each higher vitamin D quartile [40]. Similarly, Al-Daghri included 266 subjects, 153 with type 2 diabetes and 113 healthy controls, aged 26–80 [41]. Within the diabetic subjects, there was a significant negative correlation between serum vitamin D levels and HOMA-IR. There was also a significant negative relationship between vitamin D levels and insulin resistance within the entire sample. The associations remained significant after adjusting for differences in BMI [41]. 

Other studies using cross-sectional designs have failed to find significant relationships between vitamin D levels and insulin resistance. For example, Sheth et al. studied 912 subjects from Western India. About 50% were diabetic and the other half were not. More than 90% of the sample, both diabetics and non-diabetics, had vitamin D deficiencies. No significant relationship between serum vitamin D and insulin resistance was found within the diabetics or within the control participants [42]. Similarly, Al-Shoumer et al. studied 69 patients with type 2 diabetes and 60 matched normal control subjects [43]. Both groups tended to have deficient levels of vitamin D. The scientists found no relationship between serum vitamin D levels and insulin resistance. 

Clearly, correlational research has produced mixed results. It appears that a majority of investigations have shown that as serum vitamin D levels increase, insulin resistance tends to decrease. Part of this majority could be a result of multiple publications using the same data sets, therefore generating very similar findings. Additionally, some correlational studies did not adjust for potential confounding variables, which could influence results significantly. 

### 4.2. Supplemental Vitamin D Interventions and Insulin Resistance 

Many studies have investigated the effect of supplemental intake of vitamin D on insulin resistance and similar metabolic outcomes [44]. There are at least three reasons supplement vitamin D has received significant attention in the literature. First, supplemental vitamin D is easy to administer. Second, the dosage of supplemental vitamin D can be controlled precisely. Third, consumption of vitamin D via food has little effect on serum concentrations [45,46], so supplemental vitamin D must be administered to raise serum levels significantly. 

The results of the present study showed that the odds of being insulin-resistant are much higher in those who do not take supplemental vitamin D compared to those who do (Table 5). Of course, adults who take supplemental vitamin D may have other habits that protect them from insulin resistance, besides their consumption of extra vitamin D. 

Inconsistent with the present investigation, most research has found that supplemental vitamin D does not improve insulin sensitivity. A wide variety of protocols have been followed. For example, in a six-month double-blind randomized controlled trial (RCT), 62 adults received a large, single oral dose of 400,000 IU vitamin D_3_ or placebo. Another 200,000 IU dose was given if serum 25(OH)D was <100 nmol/L after 4 weeks. In the treatment group, serum levels of D_3_ increased from 38 to 97 nmol/L, but insulin sensitivity and glycemic control did not differ between the treatment and placebo groups [47]. 

Harris et al. conducted an RCT with 89 overweight or obese African Americans with prediabetes or early diabetes over 12 weeks [48]. Those in the treatment group received 4000 IU/day of D_3._ Mean D_3_ was approximately 40 nmol/L in the placebo group and 81 nmol/L in the intervention group. At the conclusion of the 12-week intervention, those in the treatment group experienced a decrease of 4% in insulin sensitivity, whereas those in the placebo group increased in sensitivity by 12%. The difference was significant, but in the unexpected direction. 

Combining vitamin D supplementation with a weight loss diet, Aliashrafi et al. studied 44 obese adults with vitamin D deficiency for 12 weeks using an RCT [49]. The subjects received 50,000 IU vitamin D_3_ once per week or a placebo. Significant weight was lost in both groups and serum vitamin D_3_ increased in the treatment group. However, within- and between-group differences were not significant for serum insulin, HOMA-IR, or QUICKI.

In a 24-month RCT, Rasouli studied overweight/obese adults at high risk of diabetes [50]. Mean age was 60.5 ± 10 years. Those in the treatment group received 4000 IU of vitamin D_3_ daily or a placebo. After 2 years of treatment with the supplement, there were no differences between the two groups in the Disposition Index (an OGTT-derived estimate of β-cell function) or HOMA-IR. 

Similarly, in a 24-week investigation, Ryu et al. enrolled 158 Korean patients with type 2 diabetes to participate in an RCT [51]. A total of 129 completed the study. All participants had low vitamin D levels to begin the study (<20 ng/mL). Those in the treatment group received 1000 IU daily of cholecalciferol and 100 mg of elemental calcium administered twice daily. Those in the treatment group completed the study with a mean of 30.2 ng/mL serum vitamin D, whereas those in the placebo group ended with 15.6 ng/mL. After the 24 weeks, there were no differences in HbA1c or HOMA-IR. 

In general, failure to find a significant treatment effect of supplemental vitamin D on insulin sensitivity, which differs in concept from the present findings, could be a result of several factors. First, the present study included a wide range of ages, with subjects as young as 18 and as old as 80 years. Few RCT have included participants covering such a large age range. Second, most intervention studies have focused on adults with low levels of vitamin D, whereas the present study included adults with significant variation in their vitamin D levels, some low and some high. Finally, intervention studies have treated participants with vastly different doses of vitamin D over widely differing durations. The present study was not an intervention study, so “existing” or “unmanipulated” vitamin D levels were used. Each of these factors, or a combination of these factors, or other reasons, could explain why most intervention studies have not shown a treatment effect, whereas the present study showed a dose–response association between serum levels of vitamin D and insulin resistance. 

Some RCT have shown improvement in insulin resistance compared to controls. For example, Niroomand et al. conducted a double-blind investigation with 162 patients with prediabetes [52]. Only 83 finished the 6-month study. High-dose supplemental vitamin D caused the treatment group to have significantly higher serum vitamin D levels (36 ng/mL vs. 16 ng/mL) than the controls. Fasting glucose and 2 h OGTT did not differ between the groups. However, HOMA-IR was significantly lower in the treatment group at 6 months. A very high subject dropout rate could have influenced the results. 

In an RCT, 81 South Asian women with insulin resistance and vitamin D deficiency were treated with 4000 IU/d or a placebo in a double-blind format [53]. The intervention lasted 6 months. Subjects each had serum vitamin D levels < 50 nmol/L. After 6 months, there were significant differences between the treatment and placebo groups showing improved insulin sensitivity and fasting insulin levels in those receiving vitamin D. 

Another RCT used 92 obese adults at elevated risk for diabetes [54]. Subjects were administered either 2000 IU/d of vitamin D, or calcium, or vitamin D and calcium, or double placebo. After a glucose tolerance test, the disposition index was boosted in the vitamin D group and diminished in the placebo group [54]. 

### 4.3. Vitamin D Levels and Risk of Developing Insulin Resistance or Diabetes over Time

Prospective cohort studies have been conducted to determine risk of type 2 diabetes based on vitamin D intake or serum levels. Results have varied. For example, according to the Nurses’ Health Study, with 83,779 women participants, there were 4843 incident cases of type 2 diabetes over a 20-year period. A food frequency questionnaire (FFQ) was employed to determine vitamin D intake. There was no association between total vitamin D intake and incident diabetes. However, comparing the highest to lowest categories of total vitamin D intake showed that risk of developing diabetes was significant and 13% lower in those with the highest supplemental vitamin D intake compared to those with the lowest intake [55]. 

The Tromso investigation studied 4157 adults prospectively for approximately 11 years. A total of 183 developed type 2 diabetes. Based on quartiles of serum vitamin D, risk of developing vitamin D was not significant after adjusting for BMI [56].

The Ely prospective study included 524 randomly selected nondiabetic men and women. Subjects were followed for 10 years. Baseline vitamin D levels were inversely related to 10-year risk of hyperglycemia, 2 h glucose, insulin resistance, HOMA-IR, and a metabolic syndrome Z-score, after adjusting for differences in covariates [57]. 

In a literature review by Mitri et al., eight prospective cohort studies were evaluated using a meta-analysis. Results showed that adults in the highest category of plasma vitamin D (>25 ng/mL) had a 43% lower risk of developing type 2 diabetes compared to subjects in the lowest category (<14 ng/mL) [36].

### 4.4. Application of the Findings

Results of the present investigation revealed significant inverse associations between serum levels of vitamin D and insulin resistance. Dietary and supplemental vitamin D were also predictive of insulin sensitivity. Although serum, dietary, and supplemental vitamin D levels were each related significantly to insulin resistance in this representative sample of U.S. adults, cross-sectional relationships cannot confirm cause-and-effect. In short, although the correlations were significant and some were dose–response and strong, concluding that higher levels of vitamin D will reduce insulin resistance would be over-stepping the scope and design of the present study. 

### 4.5. Mechanisms Associated with Vitamin D and Insulin Resistance 

In their review of the literature, Szymczak-Pajor et al. discussed several potential mechanisms that help explain the relationship between vitamin D levels and insulin sensitivity [58]. In general, findings indicated that vitamin D may be a controller of insulin secretion, Ca^2+^ levels, and the health of pancreatic cells. Multiple investigations have shown that low levels of vitamin D may lead to reduced glucose-mediated secretion of insulin in rodent pancreatic cells [59,60,61,62,63]. Additionally, multiple studies have shown that glucose-mediated production of insulin may be reestablished, as a result of supplementation of vitamin D [59,60,64]. Several clinical investigations [65,66,67] suggest that vitamin D supplementation is predictive of enhanced insulin secretion, but some studies have not confirmed this association [68,69].

A number of findings suggest that vitamin D modifies immune system performance and has significant anti-inflammatory properties [70,71,72,73,74]. Vitamin D seems to decrease fat tissue inflammation by impacting leukocyte infiltration, development of adipocytes [75,76], and by decreasing release of pro-inflammatory cytokines [58]. Bioactive vitamin D also seems to reduce the activation of mitogen-activated protein kinases (MAPK) and NF-kB signaling pathways, preventing transcription of pro-inflammatory genes [58]. Research also shows that vitamin D is a possible negative modulator of the production of pro-inflammatory cytokines [70,73], reducing C-reactive protein (CRP), interleukin 6 (IL-6), and tumor necrosis factor (TNF) [77].

Overall, several investigations indicate that inadequate levels of vitamin D may hasten the development of insulin resistance. A clearer knowledge of the vitamin D molecular contribution associated with insulin signaling may lead to new therapeutic techniques that could help reduce the risk of developing insulin resistance and related conditions.

### 4.6. Weaknesses and Strengths of the Investigation 

This study had several weaknesses. The main shortcoming was that the investigation was built on a cross-sectional design. Therefore, causal inferences cannot be employed. Additionally, only one outcome measure of insulin resistance was studied: HOMA-IR. Although a good method and the most common technique for assessing insulin sensitivity, the euglycemic insulin clamp method is usually considered the gold standard. 

This investigation also had multiple strengths. Of high importance, women and men were selected randomly using a multi-stage sampling technique, making the findings reflective of the U.S. adult population. Moreover, a big (n = 6294) and diverse sample was employed, including men and women across all races and ethnic groups. Additionally, adjustments were made for differences in many possible confounding variables to minimize their impact on the findings. Lastly, three different sources of vitamin D, serum, dietary, and supplemental, were evaluated as they relate to levels of insulin resistance.

## 5. Conclusions

Given the multitude of disorders connected with insulin resistance, the potential benefits of vitamin D consumption has attracted the interest of many scientists, physicians, and epidemiologists. The present study revealed several important findings. It determined the amount of vitamin D American men and women consume in their typical diets, the quantity of vitamin D supplements they take, the distribution of serum vitamin D levels in the U.S. population, and the level of insulin resistance that is naturally associated with the vitamin D intake of U.S. adults, diet and supplemental, without outside intervention. Overall, the literature about vitamin D and insulin resistance and type 2 diabetes is not consistent. However, collectively, it appears that individuals with low concentrations of vitamin D might be helped by increasing levels of vitamin D, typically through supplementation. Although causal conclusions are outside the scope of the present study, this investigation uncovered consistent evidence indicating that greater insulin sensitivity and less resistance goes hand-in-hand with higher levels of vitamin D. Serum levels of vitamin D were most predictive of insulin sensitivity, but dietary and supplemental vitamin D were also inversely associated with insulin resistance. 

## Figures and Tables

**Table 1:** Percentile distribution of the variables representing the U.S. adult population, n = 6294.

	Percentile				
Variable	10th	25th	50th	75th	90th
Age (years)	22.43	30.44	44.15	58.25	68.66
BMI (kg/m^2^)	21.26	23.77	27.47	31.93	37.12
HOMA-IR	0.85	1.29	2.03	3.37	5.31
Body weight (kg)	57.69	66.44	78.58	93.24	108.13
Serum levels of total vitamin D (nmol/L)	36.96	51.49	68.20	86.36	105.45
Dietary intake of vitamin D (mcg per day)	0.73	1.65	3.35	5.75	9.20
Supplement intake of vitamin D (mcg per day)	0	0	0	9.98	34.56
Physical activity (min of MVPA per wk)	0	0	58.09	237.41	477.03
Energy intake (kilocalories per day)	1217	1565	1995	2558	3125
Smoking (cigarettes per day)	0	0	0	0	9.37

BMI = body mass index; HOMA-IR = homeostatic model assessment for insulin resistance. MVPA = moderate to vigorous physical activity. For the serum vitamin D variable, n = 4805 because serum vitamin D data were not collected during the 2017–2018 cycle.

**Table 2:** Mean vitamin D levels for serum, dietary, and supplement intakes across HOMA-IR quartiles.

			HOMA-IR Category				
Vitamin D	Model	Quartile 1 * n = 1582 Mean ± SE	Quartile 2 * n = 1556 Mean ± SE	Quartile 3 * n = 1583 Mean ± SE	Quartile 4 * n = 1573 Mean ± SE	F	*p*
Dietary Intake (mcg/day)	1	5.06 ^a^ ± 0.16	4.85 ^a^ ± 0.17	4.52 ^b^ ± 0.13	4.37 ^b^ ± 0.10	8.1	0.0001
	2	4.89 ^a^ ± 0.26	4.75 ^a,b^ ± 0.26	4.53 ^b,c^ ± 0.24	4.35 ^c^ ± 0.24	2.9	0.0437
Supplement Intake (mcg/day)	1	0.90 ^a^ ± 0.06	0.76 ^b^ ± 0.06	0.70 ^b^ ± 0.06	0.67 ^b^ ± 0.06	3.5	0.0205
	2	0.91 ^a^ ± 0.08	0.76 ^b^ ± 0.08	0.70 ^b^ ± 0.08	0.65 ^b^ ± 0.09	3.3	0.0272
		* n = 1167	* n = 1188	* n = 1201	* n = 1249		
Total Serum Level (nmol/L)	1	69.15 ^a^ ± 1.14	66.58 ^a^ ± 1.28	63.37 ^b^ ± 1.24	57.18 ^c^ ± 1.10	30.3	<0.0001
	2	66.54 ^a^ ± 1.06	65.46 ^a,b^ ± 1.28	63.69 ^b^ ± 1.19	60.00 ^c^ ± 1.30	5.4	0.0029

* n represents the weighted sample number. For dietary and supplemental vitamin D intake, total n = 6294. Serum levels of vitamin D were not available for the 2017–2018 data collection cycle, so total n = 4805. Means with the same superscript letter, ^a,b,c^ were not statistically significant.

**Table 3:** The odds of having insulin resistance based on total vitamin D serum levels, indexed using quartiles, after controlling for the covariates, n = 4805.

	Outcome: Insulin Resistance (≥75th %, n = 1249)		
Vitamin D Source	Model	Odds Ratio	95% CI
Serum (nmol/L)			
Q1 (ref) vs. Q2–Q4	1	2.00	1.59–2.53
	2	1.54	1.17–2.04
Q1 (ref) vs. Q4	1	2.81	1.97–3.99
	2	1.67	1.06–2.62

Q1 = quartile 1, etc. Ref. = reference group. Weighted n for each quartile was: Q1 (n = 1167), Q2 (n = 1188), Q3 (1201), Q4 (n = 1249). Insulin resistance was defined as HOMA-IR ≥ 75th percentile. Q1 included serum vitamin D levels from 7.8–51.49 nmol/L. Q2 included 51.50–68.20 nmol/L, Q3 included 68.21–86.36 nmol/L, and Q4 was ≥86.37 nmol/L. Adults with the lowest levels of serum vitamin D (quartile 1) were compared to those with higher levels (quartiles 2–4) in the first two models. Q1 (ref) vs. Q4 indicates that only the two extreme quartiles of serum vitamin D were compared. For model 1, year, age, sex, and race were controlled. For model 2, these variables and physical activity, weight, BMI, smoking, season, and energy intake were controlled.

**Table 4:** The odds of having insulin resistance based on quartiles of dietary vitamin D, not including supplements, after controlling for the covariates, n = 6294.

	Outcome: Insulin Resistance (≥75th %, n = 1573)		
Vitamin D Source	Model	Odds Ratio	95% CI
Dietary Intake (mcg/day)			
Q1 (ref) vs. Q2, Q3, Q4	1	1.13	0.95–1.35
	2	1.13	0.90–1.42
Q1 (ref) vs. Q4	1	1.20	0.99–1.46
	2	1.14	0.86–1.50

Q1 = quartile 1, etc. Ref. = reference group. Q1 [ref] indicates that quartile 1 was the reference group. Weighted n for each quartile was: Q1 (n = 1582), Q2 (n = 1556), Q3 (1583), Q4 (n = 1573). Insulin resistance was defined as HOMA-IR ≥ 75th percentile. Q1 included dietary vitamin D levels from 0–1.65 mcg/day, Q2 included 1.66–3.35 mcg/day, Q3 included 3.36–5.75, and Q4 was ≥5.76 mcg/day. For Model l, year, age, sex, and race were controlled. For Model 2, adjustments were made for differences in year, age, sex, race, BMI, smoking, body weight, season, energy intake, and physical activity. Interpretation of the third odds ratio (1.20) is as follows: The odds of having insulin resistance were 20% higher for adults with dietary intake levels of vitamin D in Q1 compared to adults in quartiles 2, 3, and 4 combined, with year, age, sex, and race controlled. The findings were borderline significant.

**Table 5:** The odds of having insulin resistance based on supplement intake of vitamin D, after controlling for the covariates, n = 6294.

	Outcome: Insulin Resistance (≥75th %, n = 1573)		
Vitamin D Source	Model	Odds Ratio	95% CI
Supplement Intake			
No (ref) vs. Yes	1	1.42	1.11–1.80
	2	1.36	1.07–1.72

Insulin resistance was defined as HOMA-IR levels ≥ the 75th percentile. There were two supplemental intake categories. For the “No” category, n = 4800. For the “Yes” category, n = 1494. No supplement intake of vitamin D was the reference (ref) group. “Yes” meant participants took supplemental vitamin D. The range for supplemental intake was 7–865 mcg/day. For Model 1, year, age, sex, and race were controlled statistically. For Model 2, adjustments were made for differences in year, age, sex, race, BMI, smoking, body weight, energy intake, physical activity, and season. Interpretation of the first odds ratio (1.42) is as follows: The odds of being insulin-resistant were 42% higher for adults who did not consume any supplemental vitamin D compared to those who took supplemental vitamin D, after adjusting for differences in the demographic variables.

## Data Availability

All data supporting the results are available online: https://wwwn.cdc.gov/nchs/nhanes/Default.aspx (accessed on 16 March 2022).
